# Cyclonic and anticyclonic contributions to atmospheric energetics

**DOI:** 10.1038/s41598-021-92548-7

**Published:** 2021-06-24

**Authors:** Satoru Okajima, Hisashi Nakamura, Yohai Kaspi

**Affiliations:** 1 Research Center for Advanced Science and Technology, The University of Tokyo, Tokyo, Japan; 2 Department of Earth and Planetary Sciences, Weizmann Institute of Science, Rehovot, Israel

**Keywords:** Atmospheric dynamics, Physical oceanography, Climate and Earth system modelling

## Abstract

Migratory cyclones and anticyclones account for most of the day-to-day weather variability in the extratropics. These transient eddies act to maintain the midlatitude jet streams by systematically transporting westerly momentum and heat. Yet, little is known about the separate contributions of cyclones and anticyclones to their interaction with the westerlies. Here, using a novel methodology for identifying cyclonic and anticyclonic vortices based on curvature, we quantify their separate contributions to atmospheric energetics and their feedback on the westerly jet streams as represented in Eulerian statistics. We show that climatological westerly acceleration by cyclonic vortices acts to dominantly reinforce the wintertime eddy-driven near-surface westerlies and associated cyclonic shear. Though less baroclinic and energetic, anticyclones still play an important role in transporting westerly momentum toward midlatitudes from the upper-tropospheric thermally driven jet core and carrying eddy energy downstream. These new findings have uncovered essential characteristics of atmospheric energetics, storm track dynamics and eddy-mean flow interaction.

## Introduction

Transient cyclones and anticyclones are a fundamental component of the extratropical climate system, causing day-to-day weather variations. By systematically transporting heat and westerly momentum, they also act to maintain the meridional thermal structure and a midlatitude westerly jet stream in each hemisphere. Their occurrence and intensities are thus very important to climate dynamics^1,2^ and regional extreme weather events^3–6^, and therefore further investigating their behavior under the current and future climatic conditions is of great scientific and societal importance.

Since the late nineteenth century^7^, investigation of transient eddies, especially extratropical cyclones, has relied on a “Lagrangian approach”, which tracks individual moving cyclones or anticyclones^8–10^. Since gridded atmospheric datasets (analyses) became available 45 years ago, however, an “Eulerian approach” has become used widely^11,12^, as it is readily applicable also to outputs from numerical atmospheric/climate models^13^. This approach extracts sub-weekly fluctuations of such meteorological variables as pressure, temperature or wind velocity locally through digital high-pass filtering applied to their daily time series, and eddy activity is then measured locally as their temporal variance or covariance^11,12^. Regions of strong eddy activity thus measured are called “storm tracks”, and many studies have investigated the climatological seasonality of storm tracks and their interannual or decadal variability based on Eulerian statistics^14–16^. Another advantage of the Eulerian approach is its suitability to quantitative dynamical diagnoses, including the energetics based on the Lorenz energy cycle^16–18^ and the Eliassen-Palm (E-P) flux diagnosis for eddy-mean flow interaction^19–21^. The latter can, for example, illustrate horizontal propagation of wave packets and associated translation of wave-activity pseudo-momentum. The Eulerian statistics can also elucidate the dynamical distinction of an eddy-driven, subpolar westerly jet stream from a stronger thermally driven subtropical jet stream^22,23^.

The aforementioned advantages of the Eulerian approach arise from treating cyclonic and anticyclonic eddies together as deviations from the mean state, which in turn prevents us from targeting individual cyclonic and anticyclonic eddies separately. Unlike the Lagrangian approach, the Eulerian statistics thus represent unified contributions from cyclones and anticyclones, which has limited our understanding of storm tracks and associated eddy-mean flow interaction. This study is the first to evaluate and reconstruct separate contributions from both cyclonic and anticyclonic eddies onto Eulerian statistics, based on instantaneous identification of cyclonic and anticyclonic vortices.

## Results

### Identification of cyclonic and anticyclonic vortices

In the Northern Hemisphere, a cyclone (anticyclone) accompanies counterclockwise (clockwise) circulation. It is not difficult to identify its rotation center at a near-surface level, where the background westerlies and associated pressure gradient are weak. In the upper troposphere, however, identifying the rotation center is generally more difficult, because cyclonic (anticyclonic) circulations often appear as open pressure troughs (ridges) associated with a meandering westerly jet. An upper-level geopotential height field is therefore not suited for determining “centers”, because of its strong meridional gradient across the jet stream. A vorticity field may be another possibility, but the center detection actually fails due to strong shear vorticity along the westerly jet as schematically illustrated in Supplementary Fig. S1a.

To circumvent the aforementioned challenges in identifying pressure troughs and ridges (or cyclonic and anticyclonic vortices, respectively) on both sides of a meandering jet (Supplementary Fig. S1b), a new method developed here relies on curvature or curvature vorticity calculated from horizontal winds (see Methods). Supplementary Figs. S1c and S1d show snapshots of curvature fields in the upper and lower troposphere, respectively. Positive and negative curvatures correspond well to upper-tropospheric pressure troughs and ridges, respectively, and the corresponding near-surface cyclones/troughs and anticyclones/ridges as well. These troughs and ridges are represented with comparable magnitudes of curvature between the upper and lower troposphere, as an advantage of the use of curvature over other measures (Supplementary Fig. S2). In fact, relative vorticity is less effective in capturing those upper-tropospheric troughs and ridges (e.g., a ridge along ~ 165°E in Supplementary Fig. S2), due to the shear vorticity included in the full relative vorticity (Supplementary Fig. S2c). In addition, an upper-tropospheric cut-off low around [50°N, 175°W] coincides with a well-defined maximum of curvature (Fig. S1c), which cannot be captured successfully by any of the other measures (Supplementary Fig. S2). Again, this example demonstrates an advantage of using curvature, in addition to its straightforward physical meaning since its reciprocal is simply the radius of curvature and thus corresponds to the horizontal size of an eddy.

### Probability of cyclonic and anticyclonic vortices and their separate contributions to the Eulerian eddy statistics

**Figure 1:**
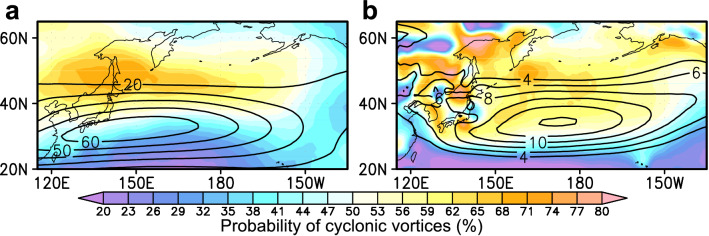
Probability of cyclonic and anticyclonic vortices. **a–b**, Climatological probability of cyclonic vortices (colors; with positive curvature) at 300-hPa (**a**) and 850-hPa (**b**) over the midwinter (24Jan) North Pacific. The probability of anticyclonic vortices can be obtained as the local residual. Black contours indicate climatological-mean U300 (**a**) and U850 (**b**) (m/s).

Climatological-mean probability of cyclonic vortices (with local positive curvature) is shown in Fig. 1 over the midwinter North Pacific. Since no threshold is set on local curvature to identify those cyclonic vortices, the local residual represents the corresponding probability of anticyclonic vortices. In the upper troposphere (Fig. 1a), cyclonic vortices are more likely to be observed to the north of the westerly jet axis, while anticyclonic vortices form more frequently to the south. A similar meridional contrast is observed across the lower-tropospheric eddy-driven westerly jet (Fig. 1b), but cyclonic vortices are often observed also around the jet axis. Contrastingly, the high probability of anticyclone vortices extending zonally around 20° − 25°N corresponds to the near-surface subtropical high-pressure belt. The lower-tropospheric statistics are overall consistent with previous results from Lagrangian tracking^9,12^.

**Figure 2:**
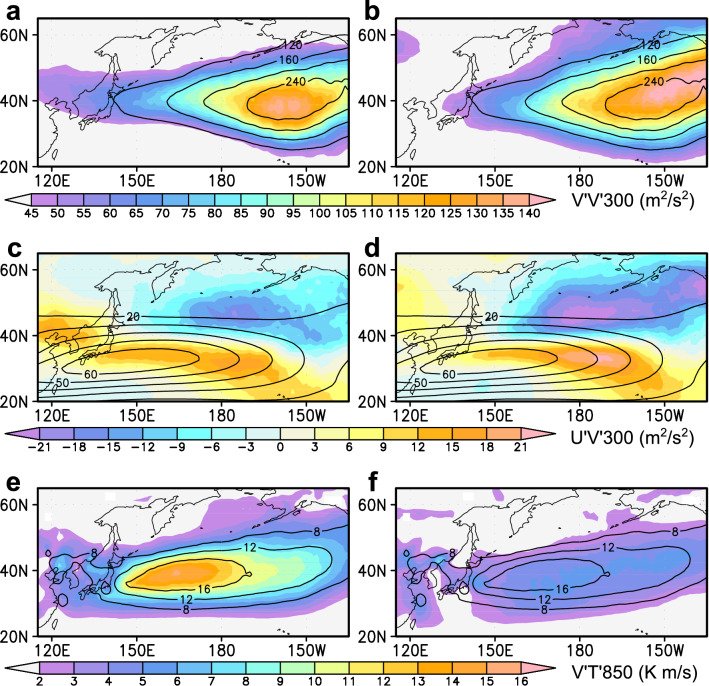
Separated contributions from cyclonic and anticyclonic vortices to Eulerian statistics. **a–b**, Contributions to climatological-mean V’V’300 (m^2^/s^2^, colors) separately from cyclonic (**a**) and anticyclonic (**b**) vortices over the midwinter (24Jan) North Pacific. Contours denote climatological-mean total V’V’300 from all vortices. **c-d**, Same as in Figs. 2a–b, respectively, but for U’V’300 (m^2^/s^2^, colors). Contours denote climatological-mean U300 (m/s). **e–f**, Same as in Figs. 2a–b, respectively, but for heat flux V’T’850 (K m/s). Contours denote climatological-mean total V’T’850 (K m/s) from all vortices.

The new method allows us to evaluate contributions from cyclonic and anticyclonic vortices (or eddies) separately to the Eulerian statistics, by accumulating instantaneous contributions only at grid points where cyclonic or anticyclonic curvature is observed. As an example shown in Supplementary Fig. S3, curvature based on instantaneous unfiltered winds is used only for determining domains of cyclonic and anticyclonic vortices, and the separation of transient eddies from the background state has been achieved by the temporal filtering. Here, no threshold is set for cyclonic and anticyclonic curvature in order not to miss any circulation on the fringes of troughs and ridges. Figures 2a–b show the climatological-mean contributions from cyclonic and anticyclonic vortices, respectively, over the midwinter North Pacific to the variance of 300-hPa high-pass-filtered meridional wind fluctuations (V’V’300) as a measure of eddy activity. As in its total field, contributions to V’V’300 from the two polarities both maximize in the eastern North Pacific, although the anticyclonic contribution is slightly larger and its maximum is located slightly downstream of its cyclonic counterpart. Distribution of 300-hPa poleward flux of westerly momentum (U’V’300) by anticyclonic vortices is similar to but stronger than its cyclonic counterpart (Figs. 2c–d). Both cyclonic and anticyclonic vortices yield the equatorward flux of momentum to the north of ~ 40°N and the poleward flux to its south. These converging fluxes thus act to accelerate the westerlies to the north and downstream of the prominent jet core around [32°N, 150°E]. The momentum flux diverges northward out of the jet core region, characteristic of a thermally driven subtropical jet^22,23^. Lower-tropospheric poleward eddy heat flux (V’T’850) by cyclonic vortices (Fig. 2e) is more than twice as strong as its anticyclonic counterpart (Fig. 2f), indicative of the prominent contribution from baroclinic cyclonic vortices to heat transport.

The threshold curvature for detecting cyclonic or anticyclonic vortices is not necessarily zero as in Fig. 2. For comparison, the corresponding climatological-mean probability of cyclonic and anticyclonic vortices is shown in Supplementary Fig. S4, with the threshold curvature equivalent to the radius of curvature of 2500 km. As expected, the probability decreases substantially for the two polarities, but the spatial distribution overall resembles that with the zero threshold. Unlike the case with the zero threshold, however, the probability of anticyclonic vortices is not necessarily a mirror image of its cyclonic counterpart. Along the upper-tropospheric westerly jet, for example, the probability for both cyclonic and anticyclonic vortices is very low. This is probably because the prominent westerly jet, especially in its core region, is likely to flow steadily and barely meanders to form vortices with small radii. Contributions to the Eulerian statistics are qualitatively similar to those with zero curvature threshold, but with more striking distinctions between cyclonic and anticyclonic V’T’850 (Supplementary Fig. S5), highlighting the dominant poleward heat flux concentrated around cyclone centers, as inferred from a typical structure of extratropical cyclones^24^.

The aforementioned characteristics of the contribution from anticyclonic vortices to the Eulerian statistics revealed with curvature, including comparable or even slightly stronger V’V’300, greater U’V’300, and weaker V’T’850 relative to their counterpart for cyclonic vortices, are not well represented if relative vorticity is used in place of curvature (Supplementary Fig. S6). The discrepancies are attributable to shear vorticity, because the decomposed Eulerian statistics based on shear vorticity exhibit consistent and even stronger biases (Supplementary Fig. S7).

### Westerly acceleration by cyclonic and anticyclonic vortices

**Figure 3:**
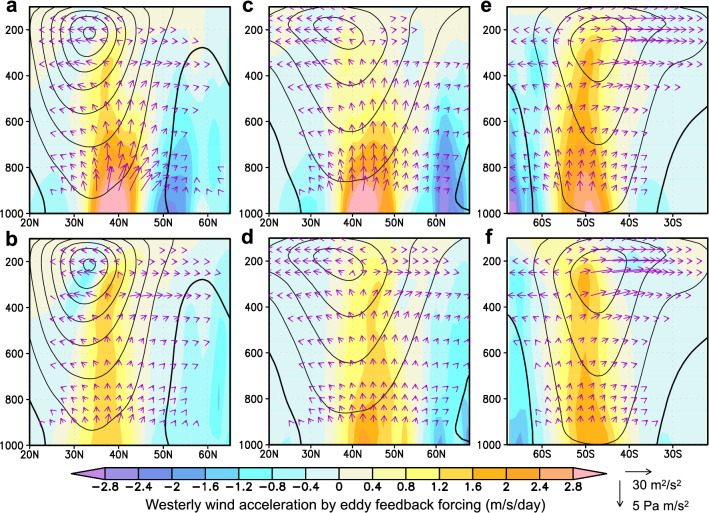
Westerly wind acceleration by transient feedback forcing evaluated separately for cyclonic and anticyclonic vortices. a-b, Meridional sections of climatological-mean net westerly wind acceleration or deceleration as transient eddy feedback forcing (colors) by cyclonic vortices (**a**) and anticyclonic vortices (**b**) for midwinter (24Jan). Quantities shown are zonally averaged for the western North Pacific [150°E − 180°]. Black contours denote climatological-mean westerly wind speed (every 10 m/s, thick line for 0 m/s). Vectors indicate extended E-P flux20 associated with cyclonic and anticyclonic vortices. (**c–d**), Same as in a-b, respectively, but for the storm track over the midwinter North Atlantic [80° − 50°W]. (**e**–**f**), Same as in a-b, respectively, but for the storm track over the midsummer South Indian Ocean [75° − 105°E].

The converging/diverging fluxes of heat and momentum by cyclonic and anticyclonic vortices imply their feedback forcing onto the climatological-mean westerlies^25,26^. To quantify this, three-dimensional eddy momentum flux convergence (divergence) is calculated as the westerly acceleration (deceleration) by eddies (see Methods for details), and its meridional sections for the western North Pacific [150°E-180°] are shown in Figs. 3a and b for cyclonic and anticyclonic eddies, respectively. Consistently with Figs. 2c–d, both cyclonic and anticyclonic vortices exert westerly deceleration (flux divergence) around the midwinter Pacific jet core (at 200-hPa) and acceleration (flux convergence) to its north through their poleward westerly momentum flux (Figs. 3a–b), although the contribution from cyclonic vortices is weaker. In fact, the westerly acceleration by cyclonic vortices occurring on the northern flank of the jet exhibits a much shallower structure (Fig. 3a). The near-surface westerly acceleration by cyclonic vortices reaches nearly 3 m/s a day, which is twice as strong as its anticyclonic counterpart and enough to replenish the climatological low-level westerlies within 3 days. This acceleration associated with the diverging upward and poleward E-P flux is due to the enhanced low-level poleward heat flux and equatorward momentum flux by cyclonic vortices. The latter diverges from the center of the Aleutian Low (AL), a semi-persistent surface oceanic low-pressure system, as marked with zero zonal wind in Fig. 3a. This diverging westerly momentum flux (or converging E-P flux) yields strong lower-tropospheric westerly deceleration near the AL center, reflecting the tendency for poleward moving cyclones to be distorted meridionally under the strong cyclonic shear of the westerlies^27^. The overall picture obtained from our analysis is that the poleward transport of westerly momentum from the upper-tropospheric core of the climatological-mean jet driven by the Hadley Cell is contributed to more by anticyclonic vortices. The transported westerly momentum is then transferred downward to maintain the near-surface westerlies around 40°N along the southern fringe of the AL, which is mainly by cyclonic vortices through their enhanced poleward heat transport. The near-surface westerly acceleration occurs also through the distorted cyclonic vortices, which is most prominent in midwinter. The result is qualitatively similar when a non-zero curvature threshold is used (Supplementary Fig. S8).

Our method to identify cyclonic and anticyclonic vortices can be applied to storm tracks over other ocean basins as well. Figures 3c–d show westerly acceleration exerted by cyclonic and anticyclonic vortices, respectively, over the North Atlantic (averaged over 80° − 50°W). In the lower troposphere, the westerly acceleration by cyclonic vortices is much stronger, while it is slightly weaker in the upper troposphere than that by anticyclonic vortices. Additionally, near-surface westerly deceleration by cyclonic vortices is striking along the southern fringe (at ~ 60°N) of the semi-permanent Icelandic Low, especially in midwinter. These characteristics are in common with the North Pacific storm track. Figures 3e–f show the corresponding westerly acceleration over the summertime South Indian Ocean (averaged over 75° − 105°E), where a distinct subpolar eddy-driven jet forms at ~ 45°S. This situation resembles that over the summertime North Pacific. Lower-tropospheric westerly acceleration by cyclonic vortices is much stronger than by anticyclonic vortices, while the contributions from cyclonic and anticyclonic vortices are comparable in the upper troposphere. These features are consistent with the two Northern Hemispheric oceanic storm tracks. Over the summertime South Indian Ocean, poleward westerly momentum flux in the upper troposphere from the subtropics into the midlatitude jet core is striking for both cyclonic and anticyclonic vortices.

### Energetics by cyclonic and anticyclonic vortices

**Figure 4:**
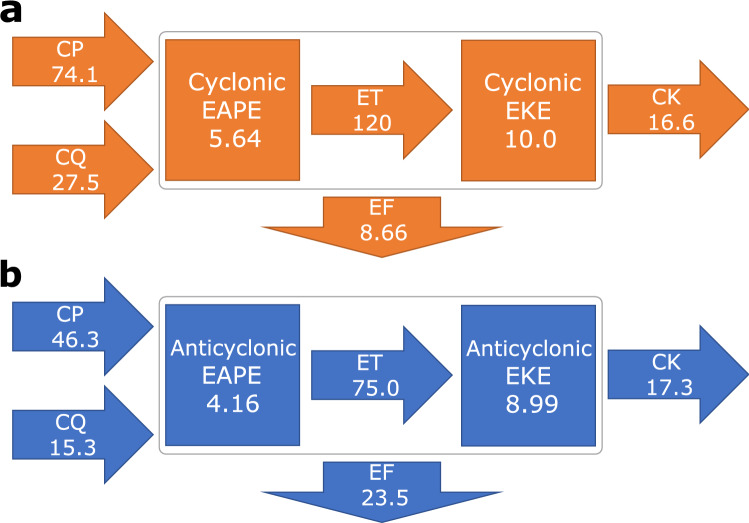
The Lorenz energetics separated into cyclonic and anticyclonic contributions. **a-b**, Climatological-mean energy budget averaged over the midwinter (24Jan) North Pacific [130° − 130°W, 20° − 65°N] for cyclonic (**a**) and anticyclonic (**b**) vortices. CK denotes the barotropic energy conversion (or KE conversion) into the background flow, CP the baroclinic energy conversion (or APE conversion) from the background flow, CQ the APE generation through diabatic processes, ET energy transfer from EAPE to EKE, and EF the energy inflow or outflow by energy fluxes through lateral boundaries of the domain. EAPE and EKE are in unit of EJ (= 10^18^ J), while CK, CP, ET and EF in unit of TW (= 10^12^ W). All the terms are integrated vertically from the surface to 100-hPa. The figure was created with Inkscape v1.0.1 (https://www.inkscape.org).

Furthermore, the decomposed Eulerian statistics can be utilized for evaluating the cyclonic and anticyclonic contributions to the Lorenz energy cycle. The atmospheric energetics for the midwinter Northern Pacific (Fig. 4) reveals that anticyclonic EKE accounts for ~ 45% of the total EKE, indicating that anticyclones are almost as important as cyclones in the midlatitude energetics. Reflecting their baroclinic structure, the ratio of EAPE to EKE for cyclonic vortices is somewhat higher than anticyclonic vortices.

The potential energy conversion (CP) to cyclonic vortices from the baroclinic background westerlies is greater by ~ 60% compared to anticyclonic vortices, while cyclonic and anticyclonic vortices contribute comparably to the barotropic kinetic energy conversion (CK) in maintaining the westerly jet stream. Both cyclonic and anticyclonic vortices contribute positively to EAPE generation through diabatic heating (CQ). A striking feature in Fig. 4 is the predominant role of anticyclonic vortices in carrying eddy energy downstream (EF) out of the Pacific storm track.

Horizontal distributions of the CK and CP terms are overall similar between cyclonic and anticyclonic vortices (Supplementary Fig. S9). Compared to cyclonic vortices, however, anticyclonic vortices give up slightly more EKE to the background jet stream just to the south of its exit, while gaining less EKE in the jet core region. Thus, the net CK to the background westerlies from anticyclonic eddies is slightly larger especially in the upper troposphere (Supplementary Figs. S10a–b). The cyclonic CP is much stronger especially over the western North Pacific (Supplementary Figs. S9c–d), where cyclonic development is promoted along a prominent oceanic frontal zone that acts to reinforce the near-surface baroclinicity in addition to abundant moisture supply from the warm Kuroshio Extension^23^. The differences between the cyclonic and anticyclonic CP are found mainly in the lower and mid-troposphere (Supplementary Figs. S10c–d). The anticyclonic CP is concentrated in the upper troposphere and actually it is comparable to the cyclonic CP when integrated only in the mid- and upper troposphere (Supplementary Fig. S11). These energetic features are overall consistent with the results in Fig. 3.

## Discussion

In this study, the conventional Eulerian statistics and Lorenz energy cycle are decomposed into the contributions from cyclonic and anticyclonic vortices based on the separate identification of these two types of vortices. This gives new insights for understanding of storm track dynamics and eddy-mean flow interaction, especially the distinct roles of cyclonic and anticyclonic vortices in maintaining the mean westerlies, in addition to the atmospheric energetics. The novel approach used here allows us to expand the knowledge about storm tracks obtained thus far based solely on either the Eulerian statistics or Lagrangian tracking. The latter has been applied almost exclusively to near-surface cyclones, but our approach suggests that roles of anticyclones should not be overlooked. Moreover, our approach allows separating the atmospheric energetics into their cyclonic and anticyclonic contributions, pointing to the important role of anticyclones in the overall energy budget and energy conversion.

Our new method can lead to identification of distinct roles, if any, of cyclonic and anticyclonic eddies in causing the counterintuitive observed midwinter suppression of the North Pacific storm track activity^14,15^. Likewise, dynamics of the annular modes over the Northern and Southern Hemispheres^28^, the baroclinic annular mode^29^, and blocking highs^30–32^ can also be addressed through our new unified approach between the Lagrangian and Eulerian perspectives. The same will be the case for output of climate models, including future climate projections and large ensemble simulations, in which changes in positions and activity of storm tracks have been intensively studied^33–36^.

Previous studies have examined the observed trend^37^ and future change^38–40^ in atmospheric energetics based on the Lorenz energy cycle. These studies helped understand climate change from an energetic point of view, into which the present study can give a new insight. Furthermore, the separation of cyclonic and anticyclonic contributions to the atmospheric energy cycle can be useful for the validation of the climate model simulations, providing us with a more phenomenological way to interpret and constituting another constraint for the models. Recently, the effect of global warming on wind power generation, which ultimately determines the amount of wind energy that can be extracted for power generation^41^, has been investigated in the framework of atmospheric energetics^42^. Our new approach has the potential to delineate separate roles of cyclones and anticyclones in the origin of near-surface wind energy.

## Methods

### JRA-55 reanalysis

We analyzed 6-hourly global fields of atmospheric variables, including SLP and geopotential height, air temperature, wind velocity, and diabatic heating rates in the pressure coordinates, obtained from the Japanese 55-year reanalysis (JRA-55) by the Japan Meteorological Agency (JMA)^43,44^ for the period 1958/59–2016/17. The JRA-55 has been constructed through a four-dimensional variational data assimilation (4D-Var) system with TL319 horizontal resolution (equivalent to 55-km) and 60 vertical levels up to 0.1-hPa. Variables on selected pressure levels are available on a 1.25° × 1.25° grid system.

### Extracting transient eddy components

At a particular grid, fluctuations of a given variable with synoptic-scale transient eddies whose period is shorter than about a week have been extracted from the 6-hourly atmospheric reanalysis as its deviations from their low-pass-filtered fields with an 8-day cutoff Lanczos filter (in this paper, primes denote local deviations from the climatological mean). Local activity of those transient eddies or their fluxes is evaluated as the variance based on the sub-weekly fluctuations of meridional velocity or the covariance representing poleward eddy heat flux. Regions of high eddy activity corresponds to “storm tracks”, along which transient eddies recurrently develop. Climatological-mean fields plotted in Figs. 2 and 3 for a given midwinter day are calculated after applying a 31-day running mean to daily climatology.

### Separation of curvature and shear vorticity

Vorticity can be decomposed locally into shear and curvature terms as follows^45–47^

1 $$\zeta = - \frac{{\partial V}}{{\partial n}} + \frac{V}{{R_{S} }}$$

where *V* denotes scalar wind speed, *n* the direction perpendicular to the flow, and *Rs* the radius of curvature. The first and second terms of the RHS in Eq. (1) represent shear vorticity and curvature vorticity, respectively, which can be calculated as

2 $$- \frac{{\partial V}}{{\partial n}} = - \frac{1}{{V^{2} }}\left( { - uvu_{x} - v^{2} v_{x} + u^{2} u_{y} + uvv_{y} } \right)$$

3 $$\frac{V}{{R_{S} }} = \frac{1}{{V^{2} }}\left( { - uvu_{x} + u^{2} v_{x} - v^{2} u_{y} + uvv_{y} } \right)$$

where *u* and *v* denote local zonal and meridional wind velocities, respectively, and subscripts *x* and *y* partial derivatives in the zonal and meridional directions, respectively. The curvature, defined as

4 $$\kappa _{2} \equiv \frac{1}{{R_{S} }} = \frac{1}{{V^{3} }}\left( { - uvu_{x} + u^{2} v_{x} - v^{2} u_{y} + uvv_{y} } \right)$$

can be derived from the definition of curvature of a two-dimensional curve implicitly represented by $\psi \left( {x,y} \right) = c$;

5 $$\kappa _{2} \equiv \frac{{\left| {\begin{array}{*{20}c} {\psi _{{xx}} } & {\psi _{{xy}} } & {\psi _{x} } \\ {\psi _{{yx}} } & {\psi _{{yy}} } & {\psi _{y} } \\ {\psi _{x} } & {\psi _{y} } & 0 \\ \end{array} } \right|}}{{\left( {\psi _{x}^{2} + \psi _{y}^{2} } \right)^{{3/2}} }}$$

The curvature or curvature vorticity enables us to circumvent the difficulties in determining areas of cyclonic and anticyclonic circulations, because it is free from shear vorticity and thus extracts vortex circulation with a certain radius. A similar quantity, curvature vorticity multiplied by a scalar wind speed (named Eulerian Centripetal Acceleration or ECA), was utilized to track 500-hPa mobile pressure troughs^45,48^. As shown in Supplementary Fig. S2e, ECA well captures centers of upper-tropospheric troughs along a strong westerly jet, which was the purpose of contriving ECA^45^. Meanwhile, ECA is less effective in representing the center of an upper-tropospheric cut-off low than the curvature (Supplementary Fig. S1c). Another aspect of ECA is a distinct difference in its amplitude between the upper and lower troposphere (Supplementary Figs. S2e and S2f.), which is due to the direct contribution of squared wind speed. At this point, curvature is suitable for identifying three-dimensional cyclonic and anticyclonic vortices and evaluating those contributions to the atmospheric energy cycle, whereas ECA is compatible with the identification of centers of troughs at a given mid- or upper-tropospheric level. In this study, curvature is weakly smoothed by applying a 9-point horizontal smoothing (weight is 0.5 next to the center point and 0.3 at corners) twice when used for determining the direction of local circulation (cyclonic or anticyclonic).

In addition to the potential application of curvature to meteorological and climatic phenomena including cut-off lows, the decomposition of vorticity into the curvature and shear terms can be useful for other fields of geoscience, because curvature is calculated purely locally with no laborious procedures required. For example, we can distinguish and identify ocean eddies and jets along the western boundary currents, or determine the boundary of a given warm/cold core eddy. In the case of the meandering Kuroshio Extension as in Supplementary Fig. S12a, relative vorticity includes mixed contributions from the vortex and shear terms (Supplementary Fig. S12b). The curvature term better depicts eddies (Supplementary Fig. S12c), while the shear term helps us identify oceanic jets (Supplementary Fig. S12d). For example, a mesoscale cyclonic eddy associated with the meandering Kuroshio is better resolved as an isolated vorticity maximum around [32°N, 139°E], whereas shear vorticity depicts more continuous bands of positive and negative values than relative vorticity, representing the meandering Kuroshio current and its eastward Extension. This decomposition can therefore be helpful for elucidating dynamical processes involved in the maintenance and variability of the oceanic jet under the possible feedback forcing from eddies. The identification of ocean eddies through curvature and curvature vorticity based on horizontal flow fields may thus be more straightforward than, for example, the commonly used Okubo-Weiss (OW) parameter^49,50^ or identification based on sea surface height^51^.

It may be informative to show the relationship between the curvature utilized in this study and the OW parameter, which is defined as

6 $${\text{W}} = \left( {v_{x} + u_{y} } \right)^{2} + \left( {u_{x} - v_{y} } \right)^{2} - \left( {v_{x} - u_{y} } \right)^{2} = 4\left( {v_{x} u_{y} - u_{x}^{2} } \right)$$

The last equality holds for the case of horizontally non-divergent flow. The last term is related to Gaussian curvature of three-dimensional surface, since for a given surface ${\text{z}} = \psi \left( {x,y} \right)$, Gaussian curvature is defined as

7 $$\kappa _{3} \equiv \frac{{\psi _{{xx}} \psi _{{yy}} - \psi _{{xy}}^{2} }}{{\left( {1 + \psi _{x}^{2} + \psi _{y}^{2} } \right)}}$$

The numerator is clearly one fourth of the OW parameter where $\psi \left( {x,y} \right)$ represents the streamfunction. The curvature used in this study focuses on a two-dimensional isocurve, while the OW parameter focuses on a curved surface.

One should be cautious in calculating Eulerian statistics based on the vorticity decomposition. In the present study, for example, V’V’ is calculated from a high-pass-filtered field of total (not decomposed) meridional wind as shown in Supplementary Fig. S3. It might be possible to calculate eddy variance and covariance from decomposed velocities such as $v = v_{C} + v_{A} + v_{S}$, where subscripts “C”, “A”, and “S” denote velocities derived from positive and negative curvature vorticity and shear vorticity terms, respectively (e.g., $v_{C} = \frac{\partial }{{\partial x}}\left( { - \left( {\nabla ^{2} } \right)^{{ - 1}} \zeta _{C} } \right)$; $\zeta _{C}$ denotes positive curvature vorticity). However, those second (or higher) order statistics can have non-negligible contributions from “cross terms” in such a way that $\overline{{V'V'}} = \overline{{V_{C}' V_{C}'}} + \overline{{V_{A}' V_{A} '}} + \overline{{V_{S}' V_{S} '}} + \overline{{V_{C}' V_{A} '}} + \overline{{V_{A}' V_{S} '}} + \overline{{V_{S}' V_{C} '}}$, and the correlation coefficients between the decomposed velocity components may not necessarily be small. Whether such second-order statistics are dominated by contributions from the decomposed vorticity terms and those from the “cross terms” are negligible should be verified in future studies.

### Evaluating eddy feedback forcing

Feedback forcing exerted on quasi-steady background flow by eddies migrating along a storm track is estimated locally as a geopotential height tendency that could be induced through fluxes of heat and vorticity by transient eddies^25,52,53^, as follows:

$$\frac{{\partial \overline{z}}}{{\partial t}} = \frac{1}{g}\left( {\nabla ^{2} + f^{2} \frac{\partial }{{\partial p}}\left( {\frac{1}{\sigma }\frac{\partial }{{\partial p}}} \right)} \right)^{{ - 1}} \cdot \left( { - f\nabla \cdot \left( {\overline{{V^{\prime } \zeta ^{\prime } }} } \right) + f^{2} \frac{\partial }{{\partial p}}\left( {\frac{{ - \nabla \cdot \left( {\overline{{V^{\prime } \theta ^{\prime } }} } \right)}}{{ - \frac{{\partial \bar{\Theta }}}{{\partial p}}}}} \right)} \right)$$

8 $$\sigma = - \frac{{\partial \bar{\Theta}}}{{\partial p}}\frac{R}{p}\left( {\frac{p}{{p_{{00}} }}} \right)^{{R/c_{p}}}$$

In Eq. (8), an overbar denotes a variable for the background flow, which corresponds to the climatological-mean state in our practice. The feedback forcing by high-frequency transient eddies was estimated through the eddy fluxes as evaluated locally from 6-hourly fluctuations through the temporal high-pass filtering. High-frequency transients are always exerting feedback forcing onto the background state in which they are embedded. In the climatological-mean state their feedback forcing must therefore be balanced with other processes. Eddy feedback forcing as acceleration (or deceleration) of westerly winds is estimated by calculating westerly wind tendency from the geopotential tendency by assuming geostrophic balance.

### FORA-WNP30 reanalysis

We utilized daily zonal and meridional velocities of ocean currents over the western North Pacific obtained from the FORA-WNP30 reanalysis^54^ by the Japan Agency for Marine-Earth Science and Technology (JAMSTEC) and Meteorological Research Institute (MRI). The FORA-WNP30 was produced by the MRI Multivariate Ocean Variational Estimation system version of 4-dimensional variational method (MOVE-4DVAR^55^), and the data assimilation system developed by the JMA/MRI, which uses an eddy-resolving ocean model for the western North Pacific. Its horizontal resolution is 1/10° × 1/10° with 54 vertical levels (0 ~ 6300 m depth). To focus on the structure of oceanic jets and mesoscale eddies, velocity fields have been smoothed by performing a 9-point horizontal smoothing (with weights of 0.5 next to the center point and 0.3 at corners) 15 times for the snapshots plotted in Supplementary Fig. S12.

### Formulation of atmospheric energy cycle

The formulation of atmospheric energetics here is following previous studies^56,57^. Climatological-mean state is considered as a background state for high-pass-filtered fluctuations. All the terms are three-dimensionally integrated over the North Pacific domain [130°E − 130°W, 20° − 65°N] and between specified vertical levels. The residue corresponds mainly to the dissipation of EKE with contributions from interactions between high-frequency eddies and low-frequency variabilities.

## Supplementary Information

Supplementary Information.

## Data Availability

The JRA-55 atmospheric reanalysis is available online in https://jra.kishou.go.jp/JRA-55/index_en.html. The FORA-WNP30 oceanic reanalysis is available online in http://www.godac.jamstec.go.jp/fora/e/index.html.
