# Supplementary material for: Cyclonic and anticyclonic contributions to atmospheric energetics
Source: Sci Rep. 2021 Jun 24;11:13202. doi: 10.1038/s41598-021-92548-7 (PMC8225635; doi:10.1038/s41598-021-92548-7)
Supplement: Supplementary file 1 — Supplementary Information. [file 41598_2021_92548_MOESM1_ESM.pdf]

Supplementary information

Cyclonic and anticyclonic contributions to atmospheric energetics

Satoru Okajima, Hisashi Nakamura, Yohai Kaspi

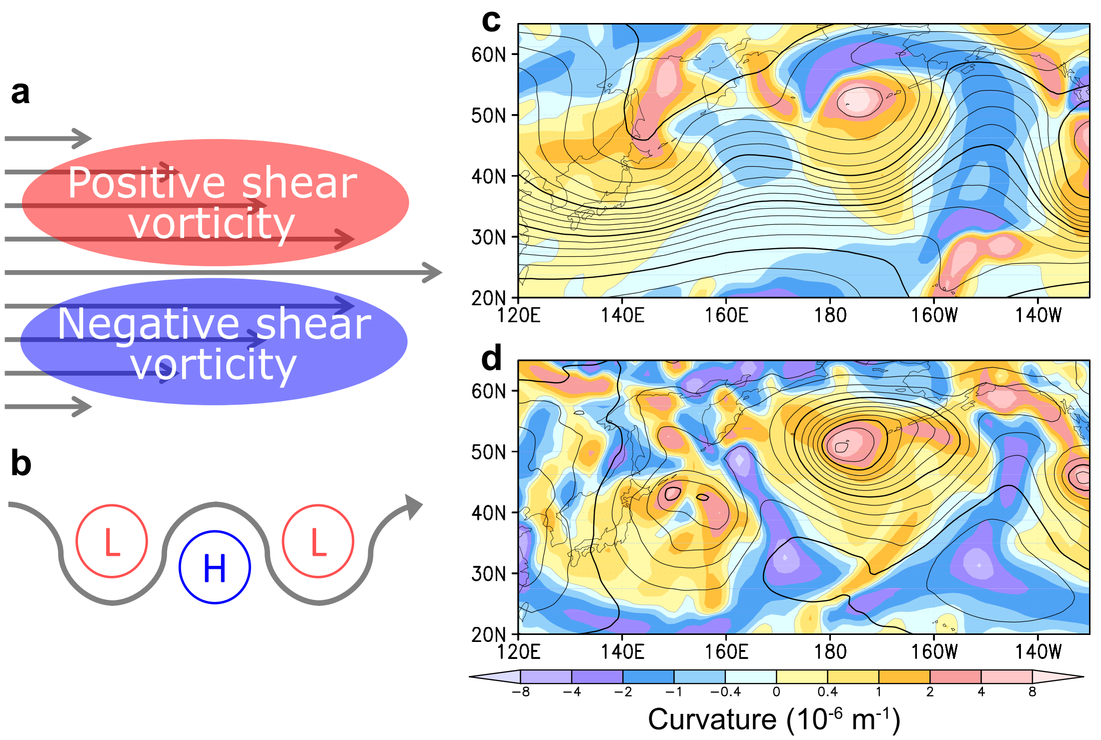

Supplementary Figure S1. Schematic diagrams of vorticity and distribution of curvature. **a**, Schematic of shear vorticity on both flanks of a straight westerly jet stream. **b**, Schematic of pressure troughs and ridges along a meandering jet stream in the Northern Hemisphere. **c-d**, Horizontal snapshots of curvature at the 300-hPa (c) and 1000-hPa (d) levels at 06UTC23Jan2017 over the North Pacific. Positive (negative) curvature signifies cyclonic (anticyclonic) circulation. Black contours denote geopotential height (every 80m and thickened for 400m) in (c) and SLP (every 6hPa and thickened for 30hPa) in (d). The figure was created with Inkscape v1.0.1 (https://www.inkscape.org).

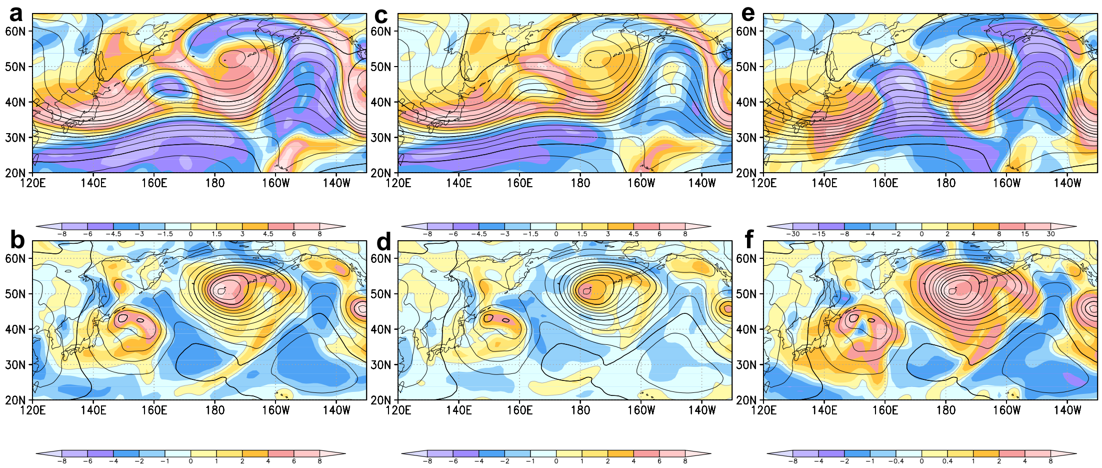

Supplementary Figure S2. Distribution of other measures.

a-b, Same as in Fig. S1c and S1d, respectively, but for relative vorticity (10^{-5} s^{-1}). **c-d,** Same as in a-b, respectively, but for shear vorticity (10^{-5} s^{-1}). **e-f,** Same as in a-b, respectively, but for ECA (10^{-4} m s^{-2}).

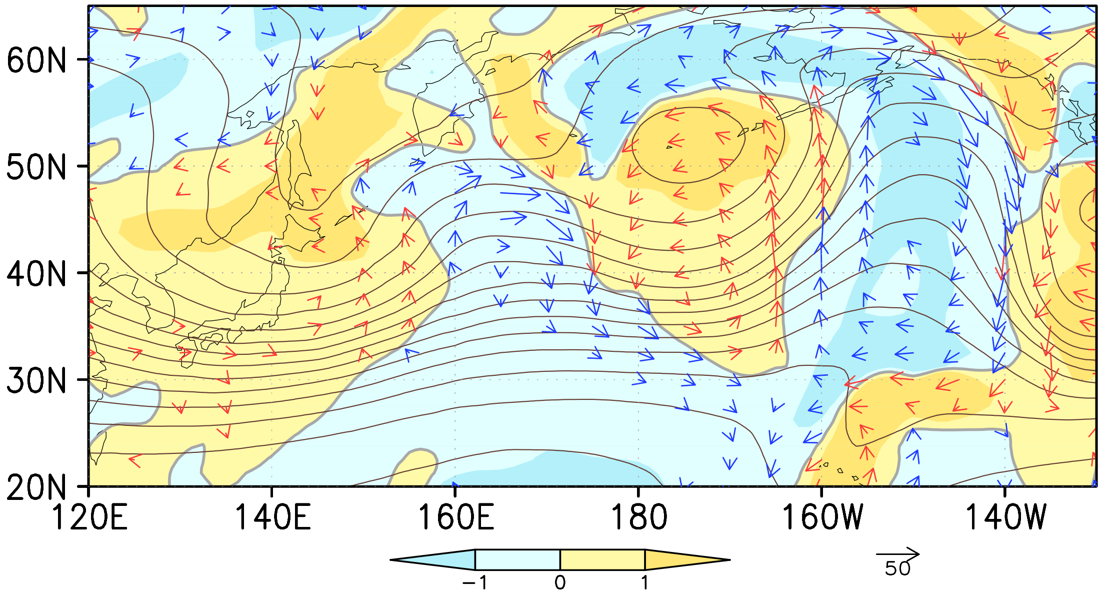

Supplementary Figure S3. Distributions of vortices and high-pass-filtered winds. Horizontal snapshot of high-pass-filtered winds (m/s as indicated) at the 300-hPa level at 06UTC23Jan2017 over the North Pacific. Red and blue arrows signify the high-pass-filtered winds associated with cyclonic and anticyclonic vortices, respectively. As in Fig. S1c, curvature (10^{-6} m^{-1}, colored) based on the corresponding unfiltered wind field indicate domains of cyclonic and anticyclonic vortices. Black contours denote the corresponding field of unfiltered geopotential height (every 100m).

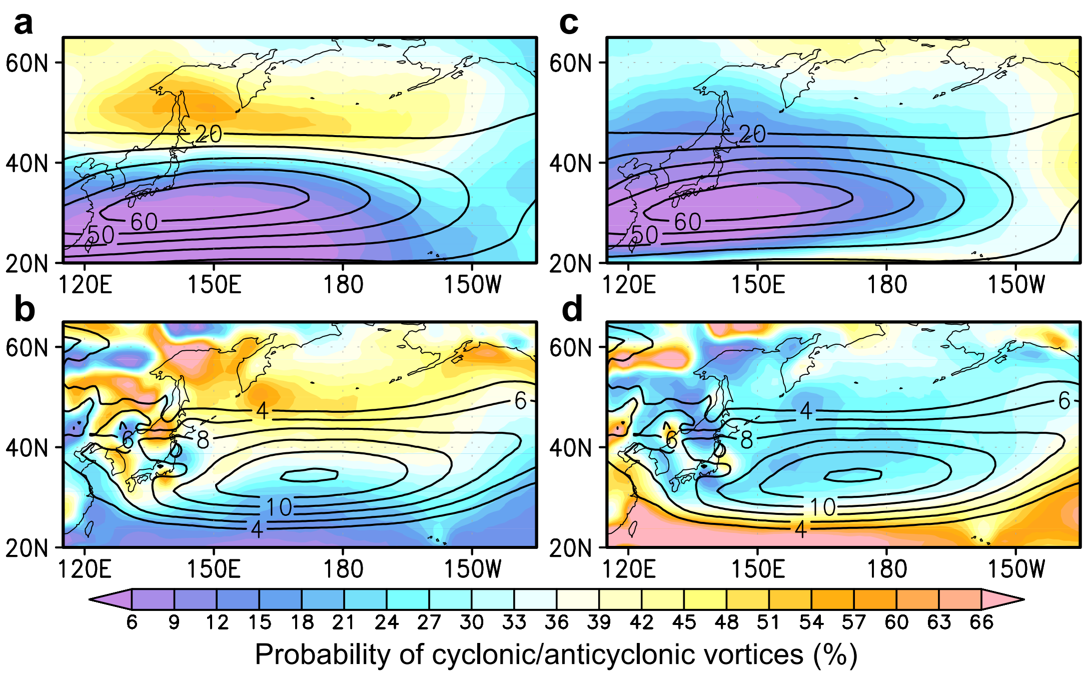

Supplementary Figure S4. Probabilities based on a non-zero curvature threshold. **a-b**, Same as in Fig. 1a-b, respectively, but for cyclonic vortices with curvature > 0.4×10^{-6} m^{-1}. **c-d**, Same as in a-b, respectively, but for anticyclonic vortices with curvature < -0.4×10^{-6} m^{-1}. These thresholds are equivalent to the radius of curvature of 2500km.

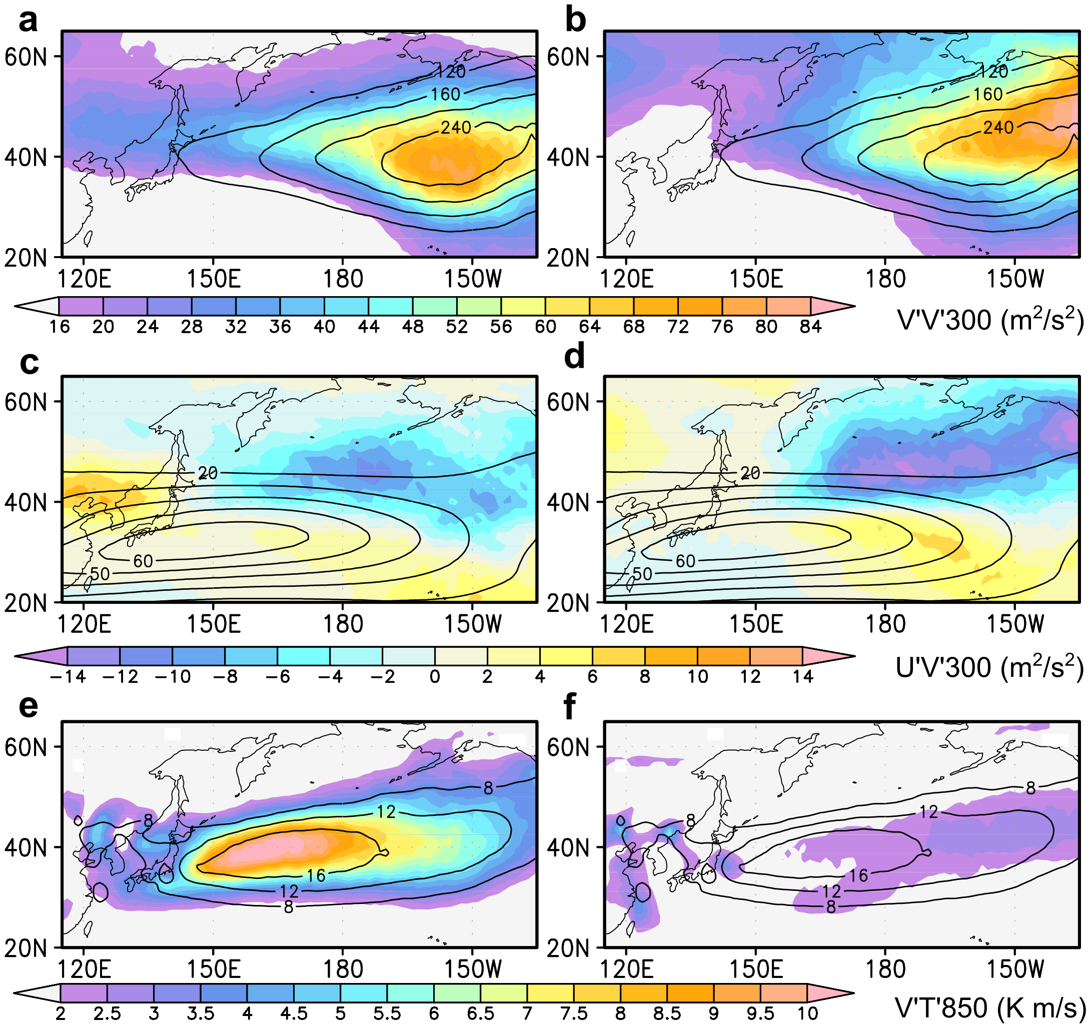

Supplementary Figure S5. Eddy statistics based on a non-zero curvature threshold. **a,c,e**, Same as in Figs. 2a, 2c and 2e, respectively, but for cyclonic vortices with curvature > 0.4×10^{-6} m^{-1}. **b,d, e**, Same as in Figs. 2b, 2d and 2f, respectively, but for anticyclonic vortices with curvature < -0.4×10^{-6} m^{-1}.

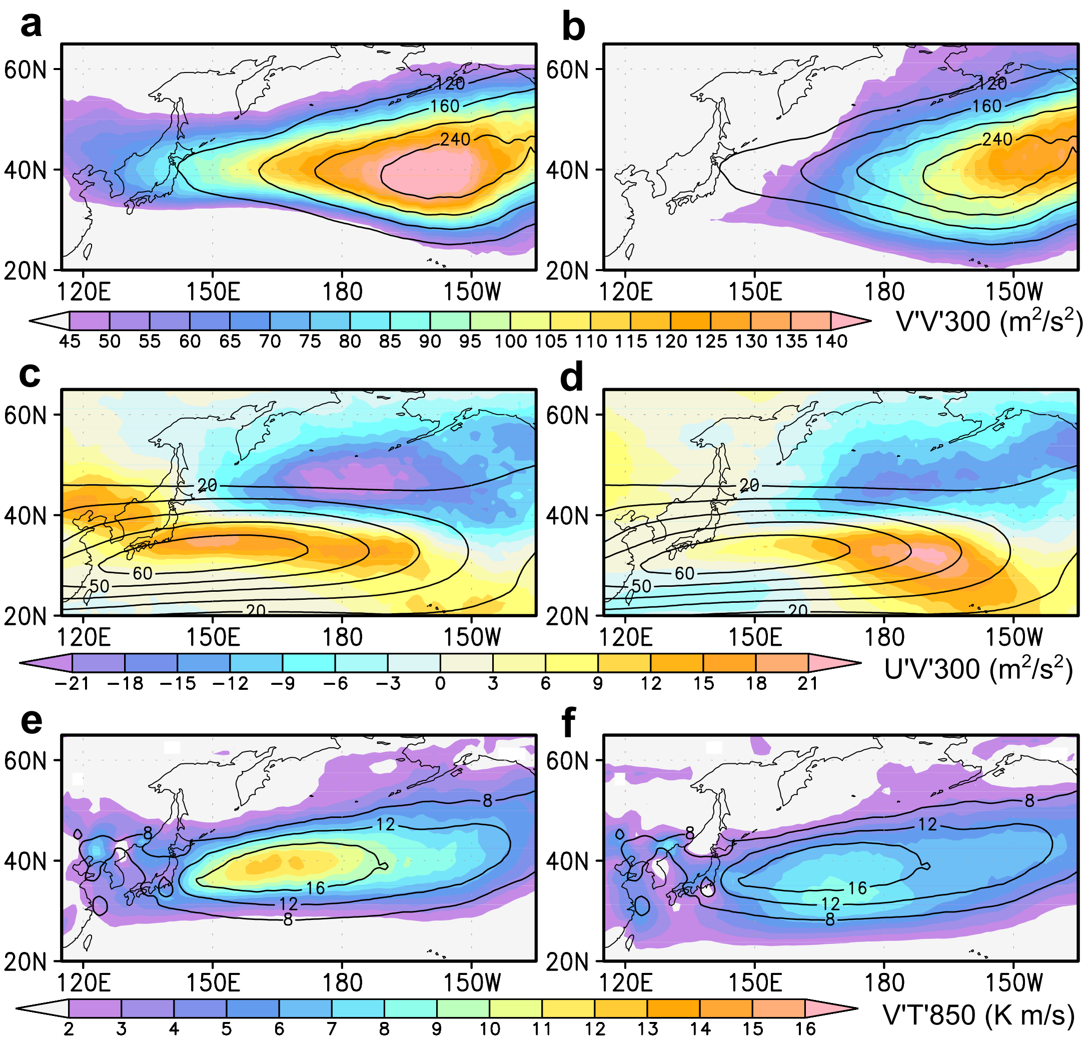

Supplementary Figure S6. Eddy statistics based on relative vorticity.

Same as in Fig. 2, but for the separated contributions to Eulerian statistics based on relative vorticity.

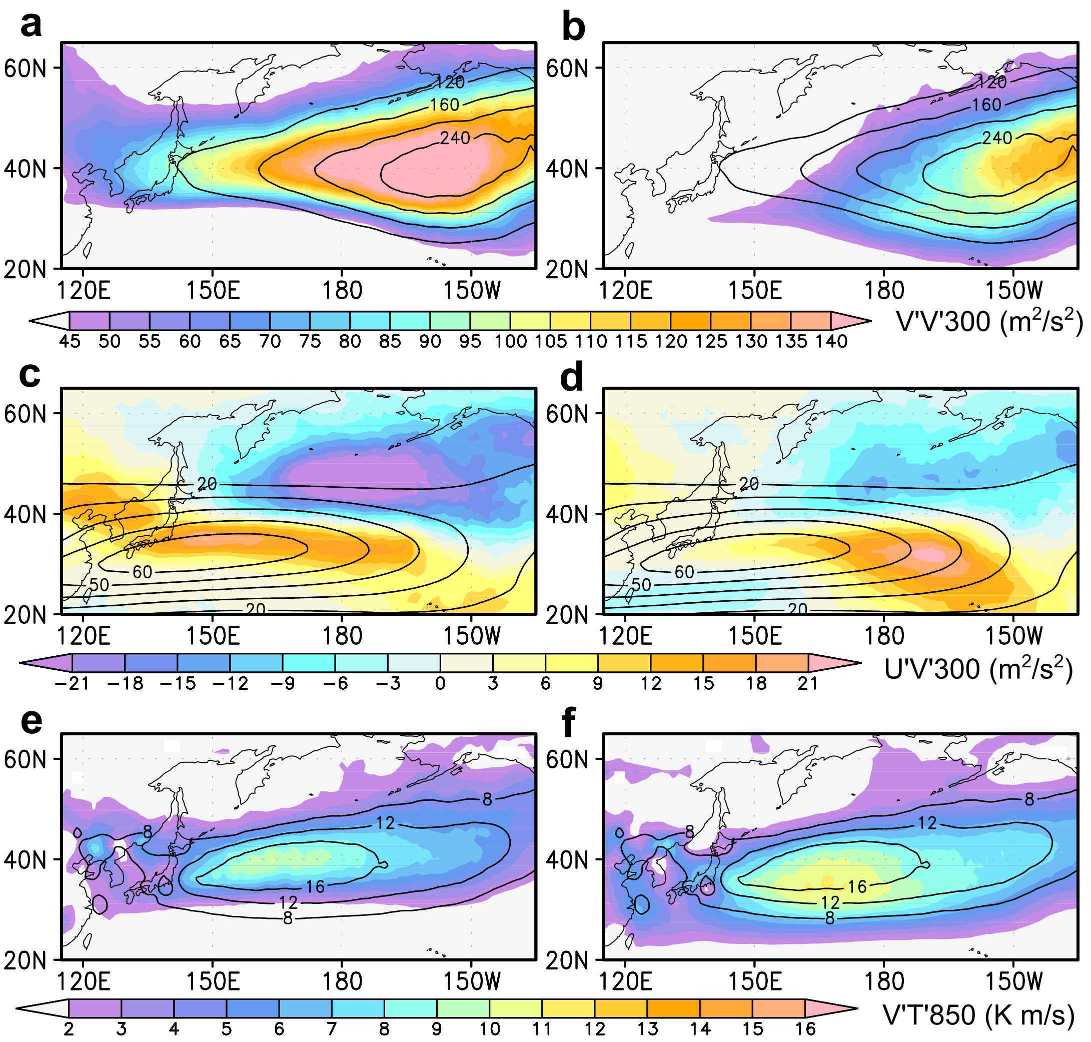

Supplementary Figure S7. Eddy statistics based on shear vorticity.

Same as in Fig. 2, but for the separated contributions to Eulerian statistics based on shear vorticity.

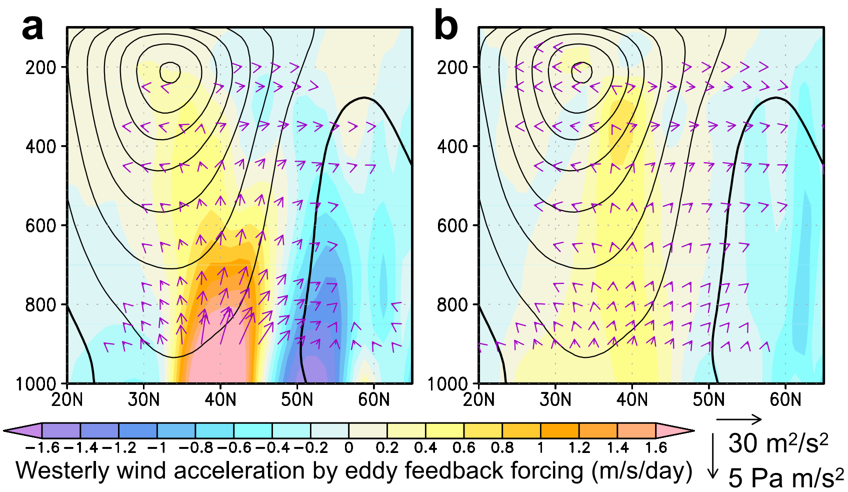

Supplementary Figure S8. Westerly wind acceleration by transient feedback forcing based on a non-zero curvature threshold.

a-b, Same as in Figs. 3a-b, respectively, but for cyclonic vortices with curvature $> 0.4\times10^{-6}$ m^{-1} (a) and anticyclonic vortices with curvature $< -0.4\times10^{-6}$ m^{-1} (b).

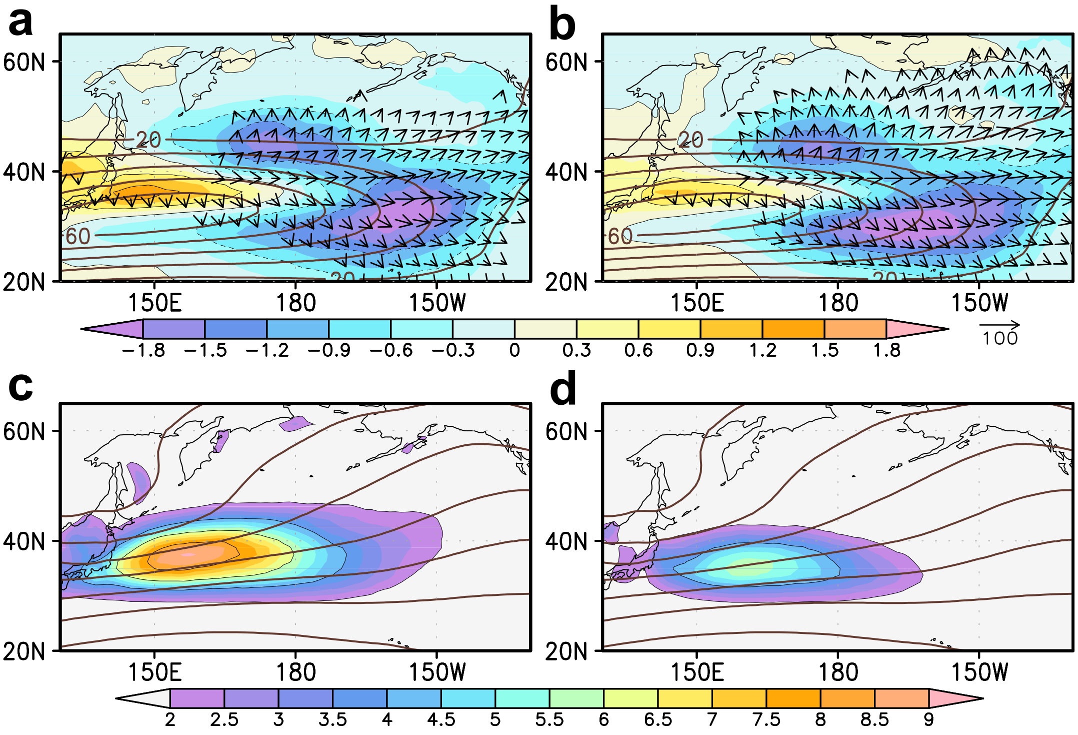

Supplementary Figure S9. Horizontal distributions of separated contributions from cyclonic and anticyclonic vortices to barotropic and baroclinic energy conversion terms. **a-b**, Contributions to barotropic energy conversion rate (CK) (W/m^2, colors) separately from cyclonic (a) and anticyclonic (b) vortices over the midwinter (24Jan) North Pacific. Contours denote climatological-mean U300 (m/s). Vectors indicate extended E-P flux[20] associated with cyclonic and anticyclonic vortices. **c-d**, Same as in a-b, respectively, but for baroclinic energy conversion rate (CP) (W/m^2, colors). Contours denote climatological-mean T700 (every 5K). All the terms are integrated vertically from the surface to 100-hPa.

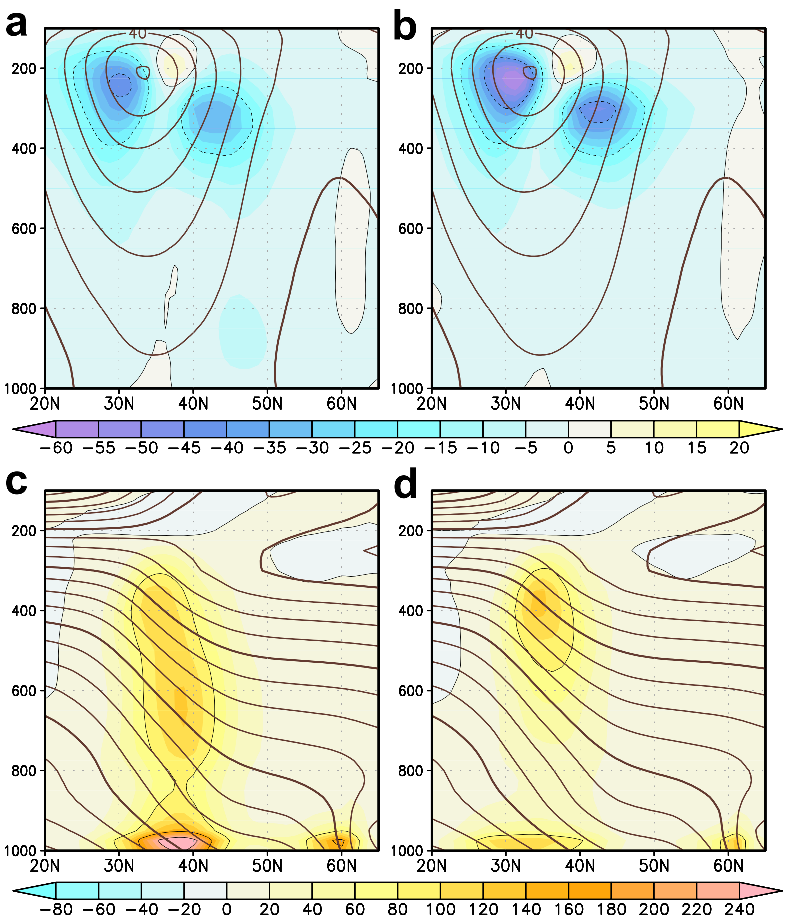

Supplementary Figure S10. Vertical structure of separated contributions from cyclonic and anticyclonic vortices to barotropic and baroclinic energy conversion terms. Meridional sections of climatological-mean barotropic energy conversion rate (CK) (10^{-5} W/kg, colors) by cyclonic vortices (a) and anticyclonic vortices (b) for midwinter (24Jan). Quantities shown are zonally averaged for the North Pacific [150°E-150°W]. Black contours denote climatological-mean westerly wind speed (every 10 m/s, thick line for 0 m/s). **c-d**, Same as in a-b, respectively, but for the baroclinic energy conversion rate (CP) averaged for the western North Pacific [150°E-180°]. Black contours denote climatological-mean temperature (every 5K).

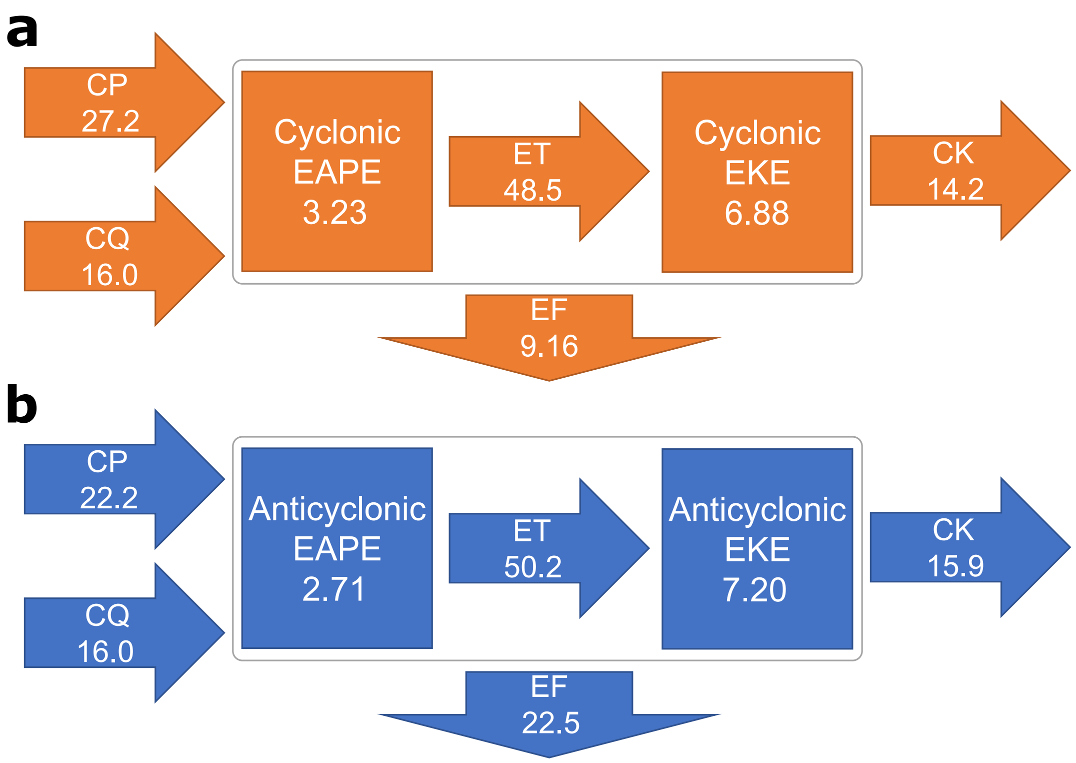

Supplementary Figure S11. The Lorenz energetics separated into cyclonic and anticyclonic contributions only in the mid- and upper troposphere. Same as in Fig. 4, but for the energy conversion/generation terms integrated vertically from 600-hPa to 100-hPa. The figure was created with Inkscape v1.0.1 (https://www.inkscape.org).

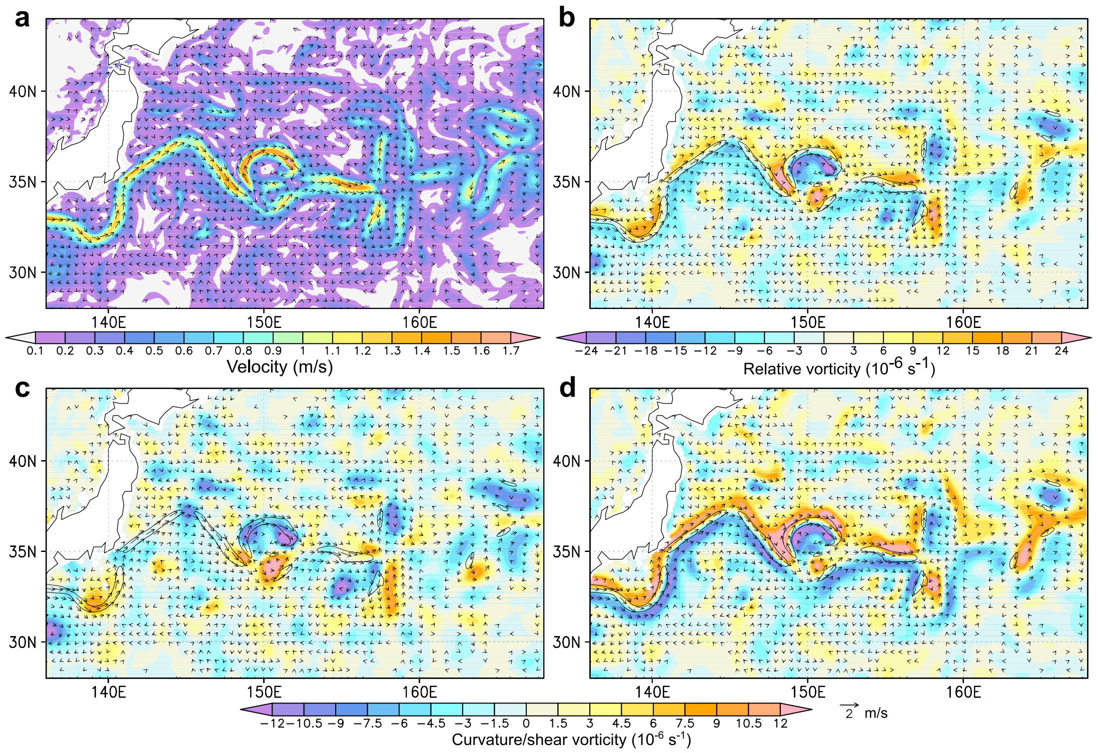

Supplementary Figure S12. Application of vorticity decomposition to meandering oceanic currents.

a, Snapshot of speed (color) and velocity (vectors as indicated) of ocean currents at 100m-depth at 00z01Jan2014. **b**, Same as in a, but for relative vorticity (horizontally smoothed). **c-d**, Same as in b, but for the curvature vorticity (c) and shear vorticity (d) terms.
